# Human Breast Milk Enhances Intestinal Mucosal Barrier Function and Innate Immunity in a Healthy Pediatric Human Enteroid Model

**DOI:** 10.3389/fcell.2021.685171

**Published:** 2021-07-13

**Authors:** Gaelle Noel, Julie G. In, Jose M. Lemme-Dumit, Lauren R. DeVine, Robert N. Cole, Anthony L. Guerrerio, James D. Campbell, Olga Kovbasnjuk, Marcela F. Pasetti

**Affiliations:** ^1^Department of Pediatrics, Center for Vaccine Development and Global Health, University of Maryland School of Medicine, Baltimore, MD, United States; ^2^Department of Internal Medicine, Division of Gastroenterology and Hepatology, University of New Mexico Health Science Center, Albuquerque, NM, United States; ^3^Department of Medicine, Division of Gastroenterology and Hepatology, Johns Hopkins University School of Medicine, Baltimore, MD, United States; ^4^Department of Biological Chemistry, Johns Hopkins Mass Spectrometry and Proteomics Facility, Johns Hopkins University School of Medicine, Baltimore, MD, United States; ^5^Department of Pediatrics, Division of Pediatric Gastroenterology, Hepatology and Nutrition, Johns Hopkins University School of Medicine, Baltimore, MD, United States

**Keywords:** enteroid, pediatric (infant), breastmilk, epithelial barrier, occludin, innate immunity, pIgR polymeric immunoglobulin receptor

## Abstract

Breastfeeding has been associated with long lasting health benefits. Nutrients and bioactive components of human breast milk promote cell growth, immune development, and shield the infant gut from insults and microbial threats. The molecular and cellular events involved in these processes are ill defined. We have established human pediatric enteroids and interrogated maternal milk’s impact on epithelial cell maturation and function in comparison with commercial infant formula. Colostrum applied apically to pediatric enteroid monolayers reduced ion permeability, stimulated epithelial cell differentiation, and enhanced tight junction function by upregulating occludin. Breast milk heightened the production of antimicrobial peptide α-defensin 5 by goblet and Paneth cells, and modulated cytokine production, which abolished apical release of pro-inflammatory GM-CSF. These attributes were not found in commercial infant formula. Epithelial cells exposed to breast milk elevated apical and intracellular pIgR and enabled maternal IgA translocation. Proteomic data revealed a breast milk-induced molecular pattern associated with tissue remodeling and homeostasis. Using a novel *ex vivo* pediatric enteroid model, we have identified distinct cellular and molecular events involved in human milk-mediated improvement of human intestinal physiology and immunity.

## Introduction

The human gastrointestinal epithelium is a selective physical and chemical barrier that separates the luminal content from the serosal compartment and inner host tissues (Zihni et al., 2016). It enables transport of electrolytes and nutrients, and provides a first line of defense against pathogens by engaging innate and adaptive mucosal immune components (Peterson and Artis, 2014). The intestinal epithelium and associated mucosal immune environment progressively develop and mature from early fetal stages through childhood by means of genetic and external signals (Torow et al., 2017; Stras et al., 2019). Human milk, rich in essential macronutrients, bioactive molecules (i.e., growth factors, antimicrobial peptides, complex oligosaccharides), and immune components including immunoglobulins, cytokines, and immune cells, supports tissue development and protects infants against infectious agents (Turfkruyer and Verhasselt, 2015). Human milk is also a source of and helps establish a healthy microbiota in infants (Ballard and Morrow, 2013). Improvement of chronic and acute diseases (e.g., necrotizing enterocolitis, inflammatory bowel diseases, and intestinal and pulmonary infections) has been attributed to breastfeeding (Barclay et al., 2009; Jantscher-Krenn et al., 2012; Oddy, 2017; Bode, 2018). Because of its countless benefits, breastfeeding has been recommended at least during the first 6 months of life (World Health Organization, 2013).

Current knowledge of the health-promoting benefits of human breast milk remains empiric or primarily descriptive, having been derived from observational or epidemiologic studies. The cellular and molecular processes underlying the effects of maternal milk in the pediatric gut and physiologic pathways involved remain ill characterized. One of the reasons for this gap in knowledge is the lack of reliable models that could recapitulate the effect of human milk on the development and maintenance of a healthy pediatric human gut and its origin in modulating systemic effects. Studies using intestinal cancer cell lines including HT-29, T84, and Caco-2 cells, or short-lived primary epithelial cells obtained from animals fail to reproduce the normal physiological responses of human intestinal epithelium (Sun et al., 2002; Lin et al., 2014; Drummond et al., 2017; Pulendran and Davis, 2020). Additionally, these immortalized cultures consist mainly of enterocytes and lack intestinal segment- and age-specificity needed for study of the complex multicellular and diverse composition of the human intestinal epithelium.

In this study, we described the establishment of an *ex vivo* 2D pediatric human enteroid model derived from intestinal Lgr5-positive stem cells and a molecular and cellular interrogation of the effects of human breast milk in the intestinal epithelium. Human intestinal enteroids (HIEs) recapitulate the crypt-villus cell axis and the segment-specific physiology (duodenum, jejunum, and ileum) of the adult human small intestine (Sato et al., 2009; Zachos et al., 2016). Technical advantages of HIEs include their capacity for long-term growth (years), which preserves donor genotype, and to grow in polarized 2D monolayers with easy access to apical and basolateral epithelial cell surfaces, which avoids the cumbersome manipulation of 3D structures (Noel et al., 2017, 2018; In et al., 2019). Herein, we present a side-by-side comparison of the molecular and cellular events affected by human milk vs. commercial infant formula in human pediatric enteroids. Outcome analyses included pediatric intestinal tissue morphology and maturation, ion and epithelial barrier permeability, antimicrobial and immune functions, and epithelial cell secretome.

## Materials and Methods

### Study Approval

Protocols for recruitment of human participants, obtaining informed consent, collecting and de-identifying biopsy samples were approved by the Johns Hopkins University School of Medicine (JHU SOM) Institutional Review Board (IRB) NA 00038329. Procedures for recruitment of mothers around delivery, obtaining informed consent, and collection and de-identification of breast milk were approved under University of Maryland School of Medicine IRB HP-00065842.

### Generation of Enteroid Monolayers

Duodenal biopsies were obtained from five healthy individuals, two pediatrics (ages 2 and 5 years) and three adults (ages 25, 27, and 81 years) through endoscopy or surgical procedure. Enteroids were generated from Lgr5-positive intestinal crypts embedded in Matrigel (Corning, United States) in 24-well plates, as previously described (Sato et al., 2011). Enteroids were expanded in growth factor-enriched media containing Wnt3A, Rspo-1, Noggin, EGF, and other nutrients (Noel et al., 2017; In et al., 2019, 2020). Multiple enteroid cultures were harvested with Culturex Organoid Harvesting Solution (Trevigen, United States), fragmented and re-suspended in expansion media and seeded (100 μl) on the inner surface of 0.4 μm Transwell inserts (Corning, United States) pre-coated with human collagen IV (Sigma-Aldrich, United States). Expansion media (600 μl) was added to the receiver plate well. Media was replenished every other day (Staab et al., 2020). Enteroid monolayer confluency was monitored by measuring TER, as previously described (Staab et al., 2020). Upon reaching confluency, monolayers were differentiated in media (DFM) free of Wnt3A and Rspo-1 for 5 days (Staab et al., 2020). All cultures were maintained at 37°C and 5% CO_2_.

### Breast Milk Preparation and Monolayer Treatment

Human colostrum was obtained from United States women 0–3 days post-delivery. Commercial infant formula powder (Similac^®^ Advance^®^ Abbot Nutrition) was resuspended in sterile distilled water following manufacturer’s instructions. Both human breast milk and infant formula suspensions were centrifuged twice (10 min each) at 3,000 × *g*. The soluble fractions were extracted, filtered (0.22 μm) aliquoted, and stored at −80°C until use. Enteroid monolayers were treated apically with 100 μl of human milk or infant formula diluted 2 or 20% in DFM. Non-treated controls were treated with 100 μl of DFM. TER was monitored daily while conducting experiments to ensure monolayer integrity.

### Dextran Permeability Assay

FITC-labeled 4 kDa dextran (Millipore Sigma, St. Louis, MO, United States; 0.01% w/v in DFM) was added to the apical side of enteroid monolayers pre-treated with 20% of human milk or infant formula. Regular DFM (600 μl) was added to the basolateral side. Basolateral media (100 μl) was sampled at 30 min, 1 and 2 h, and FITC-dextran content was measured by fluorescence intensity using an EnVision Multilabel Plate Reader (PerkinElmer, Waltham, MA, United States). Sampled volume was replenished with fresh DFM.

### Immunofluorescence Staining and Confocal Imaging

Enteroid monolayers were fixed for 40 min in 4% paraformaldehyde (Electron Microscopy Sciences, United States), washed with PBS for 10 min, permeabilized and blocked for 1 h with PBS containing 15% fetal bovine serum, 2% BSA, and 0.1% saponin, all at room temperature (RT). After washing with PBS, monolayers were incubated overnight at 4°C with primary antibodies (diluted 1:100 in PBS). Primary antibodies (Ab) against the following molecules were used: occludin [mouse monoclonal (mAb), clone OC-3F10, Thermo Fisher Scientific], TFF3 [rabbit polyclonal (pAb), Millipore Sigma], lysozyme EC 3.2.1.17 (rabbit pAb, Dako), DEFA5 (mouse mAb, clone 8C8, Millipore Sigma), and SC-pIgR (rabbit pAb provided by Dr. A. Hubbard, Johns Hopkins University School of Medicine) (Solari et al., 1989). Stained monolayers were washed with PBS (3 times, 10 min each) and incubated with secondary antibodies (diluted 1:100 in PBS) for 1 h at RT. Secondary antibodies included goat anti-mouse Alexa Fluor-488 or –568, and goat anti-rabbit Alexa Fluor-488 or –568 (all Thermo Fisher Scientific). F-actin was detected by phalloidin Alexa Fluor-633, –647, -or –568 (1:100; Thermo Fisher Scientific). Hoechst for nuclear/DNA labeling (Thermo Fisher Scientific) was used diluted 1:1,000 in PBS. After incubation, cells were washed as described above, and mounted in FluorSave reagent (Millipore Sigma). Confocal images were taken using an LSM-510 META laser scanning confocal microscope (Zeiss, Germany) and ZEN 2012 imaging software (Zeiss) or BZ-X700 fluorescence microscope (Keyence, Japan) available through the Fluorescence Imaging Core of the Hopkins Basic Research Digestive Disease Development Center. For qualitative analysis, image settings were adjusted to optimize the signal. For quantitative analysis, the same settings were used across the samples, and protein-of-interest average intensity fluorescence was analyzed using MetaMorph software (Molecular Devices, CA, United States).

### Protein Extraction, Immunoblotting, and Proteomic Analysis

Enteroid monolayers were lysed in cold lysis buffer (60 mM HEPES pH 7.4, 150 mM KCl, 5 mM Na3EDTA, 5 mM EGTA, 1 mM Na3VO4, 50 mM NaF, 2% SDS) supplemented with 1:100 of protease inhibitor cocktail (P8340, Millipore Sigma). Lysis buffer was applied to the apical surface, and cells were scraped and sonicated on ice (three times at 10 s pulses each time using 30% energy input). The lysates were centrifuged 10 min at 14,000 rpm at 4°C, and the supernatant containing soluble and membrane proteins were collected. Total protein concentration was determined using a DC protein assay (Bio-Rad, CA, United States). Proteins were separated on Novex Wedgewell 4–20% gradient Tris-glycine gels (Life Technologies, CA, United States) and transferred to nitrocellulose membranes. The following primary antibodies were used for immunoblotting: rabbit pAb anti-pIgR (Abcam), anti-SC-pIgR pAb (described above), and rabbit pAb anti-FcRn (Novus Biologicals)—all at a 1:250 dilution, and mouse mAb anti-GAPDH (clone 6C5, Abcam) at 1:1,000 dilution. Secondary antibodies included goat anti-mouse Alexa Fluor Plus-680 or –800 and goat anti-rabbit Alexa Fluor Plus-680 (Thermo Fisher Scientific). Western blots were processed using the iBind Flex device (Life Technologies, Carlsbad, CA, United States) and then imaged on an Odyssey CLx imager (LI-COR, Lincoln, NE, United States). Proteomic analysis was conducted on basolateral media of pediatric monolayers treated with human milk (*n* = 3), infant formula (*n* = 1) and non-treated control (*n* = 2) through the Mass Spectrometry and Proteomics Facility, Johns Hopkins University School of Medicine. Isotopically resolved masses in precursor (MS) and fragmentation (MS/MS) spectra were extracted from raw MS data in Proteome Discoverer software (v2.2, Thermo Scientific). All extracted data were searched using Mascot (2.5.1^1^) against the RefSeq2015 protein database containing Human and common contaminants. The following criteria were set for all database searches: Human species; trypsin as the enzyme, allowing one missed cleavage; cysteine carbamidomethylation and TMT-N-term as fixed modifications; TMT on lysine, methionine oxidation, asparagine and glutamine deamidation as variable modifications. Search tolerances were set to 8 ppm and 0.02 Da for the precursor and fragments, respectively. Peptide identifications from Mascot searches were filtered at 1% False Discovery Rate (FDR) confidence threshold, based on a concatenated decoy database search, using the Proteome Discoverer.

### Cytokines/Chemokines

Cytokines and chemokines were quantified using commercial electrochemiluminescence microarray kits (Meso Scale Diagnostic, Rockville, MD, United States) following the manufacturer’s instructions. MCP-1, GM-CSF, and IL-8 levels were reported as the amount contained in the total volume of culture supernatant collected from the apical and basolateral side of the monolayers.

### Statistics

Statistical significances were determined using the Student’s *t*-test or Mann Whitney test (in the absence of normal distribution) for comparison of two groups, or one-way-ANOVA with Šidák’s or Tukey’s post-test for comparison of more than two groups. PCA was performed by selecting PC with eigenvalues greater than 1.0 (Kaiser rule). Statistical analyses were performed using Prism software v9 (GraphPad, San Diego, CA, United States). Differences were considered statistically significant at *p*-value ≤ 0.05.

## Results

### Pediatric and Adult Enteroid Monolayers Exhibit Distinct Cell Morphology and Maturation Features

**FIGURE 1:**
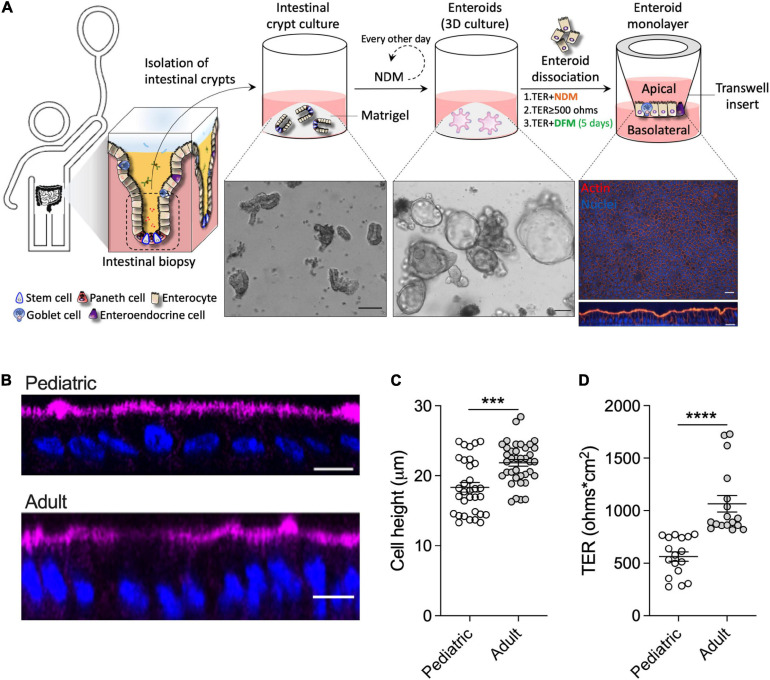
Pediatric and adult enteroid monolayers exhibit distinct maturation features. **(A)** Schematic representation of the generation of pediatric enteroid monolayers. Scale bar: brightfield images, 100 μm; immunofluorescence images (XY and XZ projections), 20 μm. NDM, non-differentiation media; DFM, differentiation media. **(B)** Confocal microscopy images (XZ projections) depicting the difference in epithelial cell height between pediatric and adult enteroid monolayers. Actin, magenta; DNA, blue. Scale bar = 20 μm. **(C)** Epithelial cell heights quantified by immunofluorescent confocal microscopy analysis (≥8 different view fields). **(D)** TER values of enteroid monolayers. Images are representative of three independent experiments **(B)**. Data shown in panel **(C)** and **(D)** represent the mean ± SEM from three **(C)** or two **(D)** independent experiments that included *n* = 8–12 enteroid monolayers/group per experiment. Each symbol represents an independent monolayer. **(B–D)** All measurements included 2 pediatric- and 3 adult-derived monolayers. **(C,D)** *p*-values were calculated by Student’s *t*-test. ***P < 0.001; ****P < 0.0001.

To mechanistically interrogate the physiological effects of human breast milk in the pediatric gut, differentiated (villus-like) enteroid monolayers were established from duodenal biopsies of healthy 2- and 5-year-old children who underwent diagnostic endoscopy (Figure 1A) at The Johns Hopkins Hospital, using methods previously described (Noel et al., 2017; Staab et al., 2020); these monolayers are hereafter referred to as 2PD and 5PD, respectively. The cell morphology, permeability, and barrier integrity of the pediatric monolayers were compared with those derived from adult duodenal tissue. Differentiated enterocytes of pediatric origin were significantly shorter than their adult counterparts as revealed by confocal microscopy images (Figure 1B) and epithelial cell height measurement (Figure 1C). Analysis of the epithelial barrier function by transepithelial electrical resistance (TER) revealed increased paracellular ion permeability in the pediatric- as compared to the adult-derived monolayers (Figure 1D).

### Human Breast Milk Improves Pediatric Epithelial Barrier Function

**FIGURE 2:**
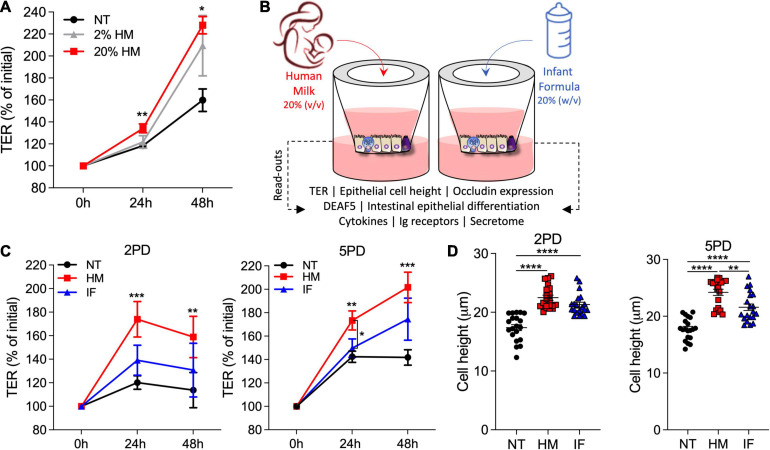
Human milk decreases ion permeability of the pediatric intestinal epithelium. **(A)** TER values of 2PD monolayers apically exposed to human milk (HM) 2 or 20% (v/v). **(B)** Schematic representation of pediatric HIE treatment and biological readouts. **(C)** TER measurement of 2PD and 5PD monolayers apically treated with 20% (v/v) of HM or 20% (w/v) of commercial infant formula (IF). **(A,C)** Mean ± SEM are shown. Data are representative of three independent experiments with *n* = 3–6 enteroid monolayers/group per experiment. *p*-values were calculated by Mann Whitney test. Unless indicated, *p*-values correspond to treated vs. non-treated (NT) controls. **(D)** Epithelial cell height quantified by immunofluorescent microscopy (≥10 different view fields). Data represent mean ± SEM of three combined experiments, each including four monolayers/group per experiment. Each symbol indicates an independent monolayer. *p*-values were calculated by one-way ANOVA with Tukey *post hoc* analysis. *P < 0.05; **P < 0.01; ***P < 0.001; ****P < 0.0001.

We next examined the effect of human breast milk (colostrum) on pediatric intestinal barrier function. Breast milk was applied to the apical side of differentiated pediatric enteroid monolayers, and TER values were monitored daily for 48 h. Monolayers exposed to human breast milk exhibited higher TER values as compared to non-treated controls (Figure 2A). A significant dose-response effect was observed with the 20% (v/v) treatment resulting in higher TER values as compared to non-treated controls (Figure 2A). This observation was consistent in multiple experiments using both 2PD and 5PD cell lines; the 20% (v/v) solution was therefore selected for subsequent experiments and examined alongside 20% (w/v) infant formula solution (Figure 2B). Human breast milk significantly and reliably increased TER levels in both 2PD and 5PD monolayers as compared to non-treated controls and remained elevated or further improved with prolonged exposure (Figure 2C). By contrast, ion permeability was modestly affected by infant formula (Figure 2C). In addition to transepithelial ion permeability by TER, paracellular molecular permeability was examined by exposing breast milk- and infant formula-treated pediatric monolayers to FITC-labeled 4kDa dextran for up to 2 h. No differences were observed in the amount of dextran recovered from the basolateral side regardless of treatment (data not shown) confirming integrity of the epithelial barrier. Finally, cell height was measured in monolayers exposed to breast milk and infant formula to discern their influence in cell morphology and growth. Both treatments increased epithelial cell height as compared to non-treated controls (Figure 2D).

### Human Breast Milk Increases the Expression of the Tight Junction Protein Occludin

**FIGURE 3:**
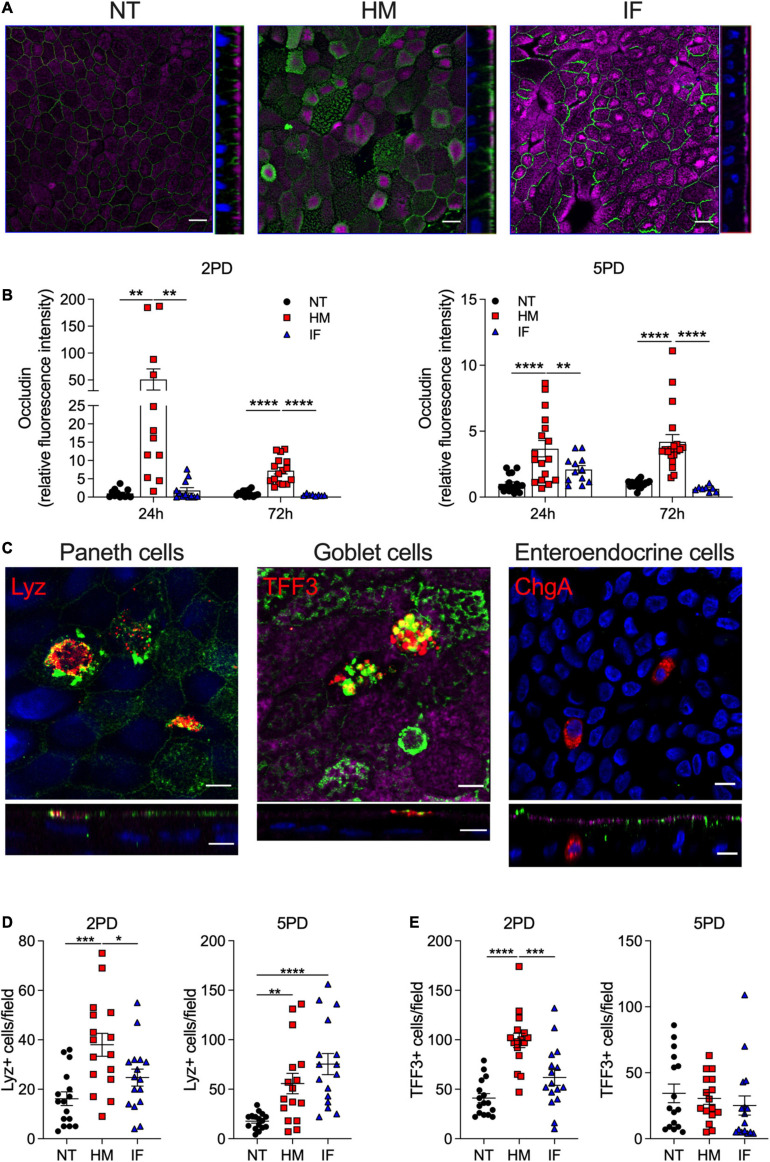
Human milk modulates occludin expression. **(A)** Confocal microscopy images (XY and YZ projections) of 2PD enteroid monolayers NT or apically treated for 24 h with HM (20%; v/v) or IF (20%; w/v). Occludin, green; actin, magenta. Scale bar = 10 μm. **(B)** Relative fluorescence intensity of occludin quantified by confocal microscopy analysis of monolayers treated with HM (20%; v/v) or IF (20%; w/v) for 24 and 72 h. Mean ± SEM are shown. Data represent three independents with *n* = 4–6 enteroid monolayers/group per experiment. Each symbol indicates an independent monolayer. *p*-values were calculated by one-way ANOVA with Šidák’s *post hoc* analysis. **(C)** Confocal microscopy images of 5PD enteroid monolayers treated with HM for 48 h. Occludin, green; lysozyme (Lyz), red; trefoil factor 3 (TFF3), red; chromogranin A (ChgA), red; actin, magenta; DNA, blue. Paneth and goblet cells, scale bar = 5 μm; enteroendocrine cells, scale bar = 10 μm. **(A,C)** Data are representative of three independent experiments with *n* = 3 enteroid monolayers/group per experiment. *p*-values were calculated by one-way ANOVA with Šidák’s *post hoc* analysis. **(D)** Number of Lyz-positive **(E)** and TFF3-positive cells quantified by immunofluorescent confocal microscopy analysis of monolayers treated with HM and IF as described in panel **(A)**. Data represent mean ± SEM of three combined experiments, each including four monolayers/group per experiment. Each symbol indicates an independent monolayer. *p*-values were calculated by one-way ANOVA with Tukey *post hoc* analysis. *P < 0.05; **P < 0.01; ***P < 0.001; ****P < 0.0001.

Maternal milk enhancement of TER values prompted us to investigate its effect on expression of tight junction (TJ) proteins, which seal the paracellular space of the intestinal epithelia and regulate passage of ions and small molecules. Occludin, a transmembrane protein of the TJ complex, was selected for this analysis as primary marker of epithelial differentiation and barrier function (Al-Sadi et al., 2011). Immunofluorescent imaging revealed occludin on the cell perimeter of all monolayers, regardless of treatment (Figure 3A). Strikingly, pediatric monolayers exposed to human milk exhibited a distinctive pattern of apical and condensed cytoplasmic vesicular expression of occludin (Figure 3A) that markedly contrasted with the perimeter-only expression of monolayers treated with infant formula. Quantitative analysis of the fluorescence intensity by confocal imaging revealed superior occludin expression in both pediatric monolayers treated with human breast milk as compared with monolayers treated with infant formula or untreated controls (Figure 3B). Of the two enteroid lines, the 2PD was the higher and more consistent responder (Figure 3B). Infant formula increased occludin expression modestly and occasionally, not reaching significance above the non-treated controls (Figure 3B). The granular occludin expression pattern induced by breast milk was observed not only in absorptive enterocytes, visible by their prominent apical brush border, but also in cells lacking brush border, which are typically secretory epithelial cell lineages such as Paneth cells, goblet cells, and enteroendocrine cells (our HIE monolayers were not induced to express M cells). To identify the specific cell types producing occludin, breast milk-treated monolayers were co-stained to detect the presence of occludin as well as lysozyme, a marker for Paneth cells, trefoil factor 3 (TFF3), a marker for goblet cells, and chromogranin A, a marker for enteroendocrine cells. Occludin granular pattern co-localized with both lysozyme and TFF3, but not with chromogranin A marker (Figure 3C). These results indicate that breast milk elevates occludin expression not only at the TJ but also in the cytoplasm and apical membrane of absorptive enterocytes as well as in Paneth cells and goblet cells. Monolayers treated with breast milk had increased number of lysozyme-expressing Paneth cells and TFF3-positive goblet cells as compared to untreated controls (Figures 3D,E); this was most evident in 2PD, suggesting a temporal/developmental stage-dependent response.

### Human Milk Increases Epithelial Cell Expression of Innate Immune Mediators

**FIGURE 4:**
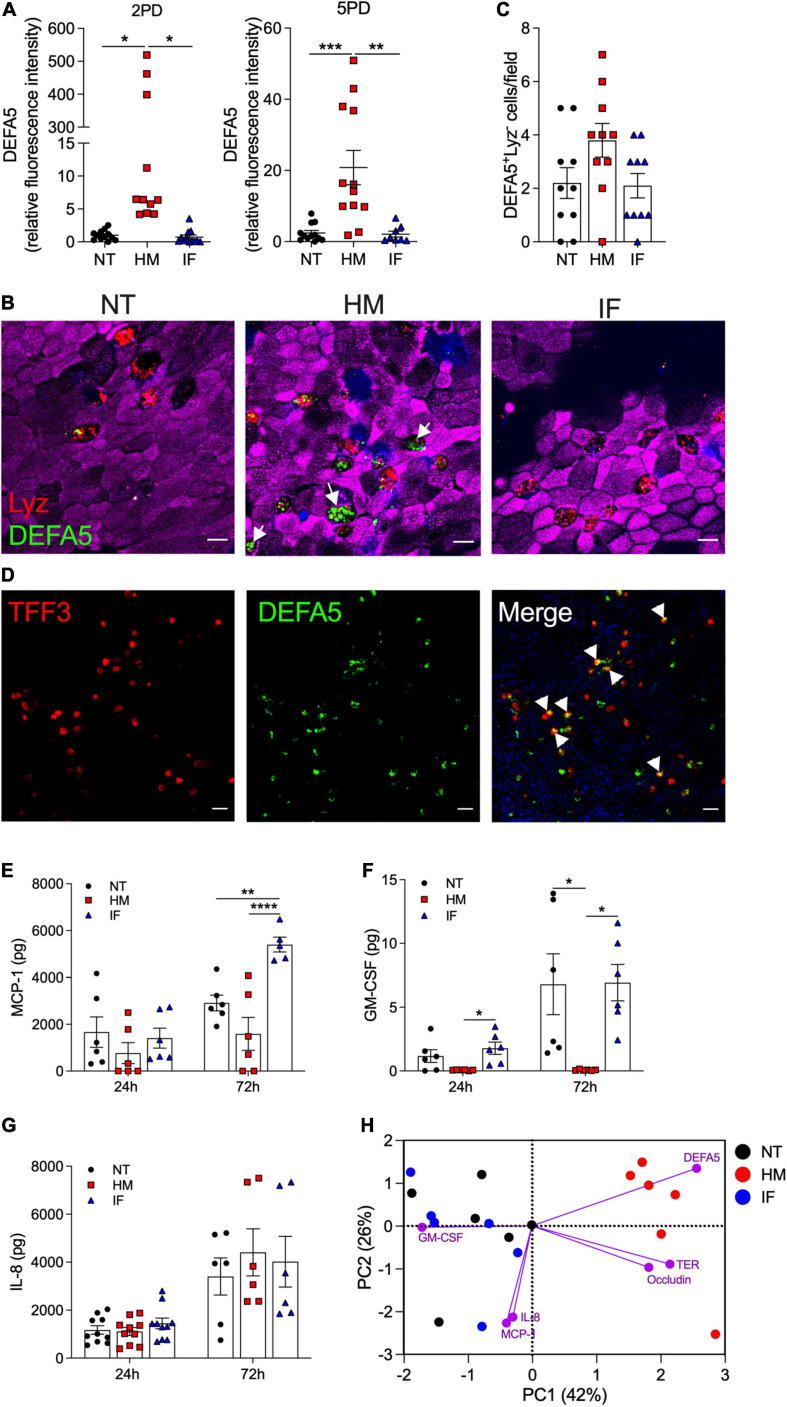
Human milk modulates epithelial innate immune function. **(A)** Relative fluorescence intensity of human DEFA5 quantified by confocal microscopy analysis of 2PD and 5PD monolayers NT or treated with HM (20%; v/v) or IF (20%; w/v) for 48 h. **(B)** Representative confocal microscopy images (XY projections) of 5PD monolayer showing localization (arrowheads) of DEFA5 in Lyz-negative cells in HM-treated monolayer and **(C)** number of Lyz-negative cells quantified by immunofluorescent confocal microscopy (≥6 different view fields). DEFA5, green; Lyz, red; actin, magenta; DNA, blue. Scale bar = 10 μm. **(D)** Representative confocal microscopy images (XY projections) of 5PD monolayer depicting co-localization (arrowheads) of TFF3 (red) and DEFA5 (green); DNA, blue. Scale bar = 50 μm. **(E–G)** Total amount of MCP-1, GM-CSF, and IL-8 in the apical media of 2PD monolayer treated with HM and IF as described in panel **(A)** for 24 and 72 h. **(H)** PCA plot from HM- and IF-treated monolayers, and NT controls for 24 h. PC, principal component. Variables analyzed: TER, occludin, DEFA5, MCP-1, GM-CSF, and IL-8. **(A,C,E–G)** Mean ± SEM are shown. Data are representative of three independent experiments with *n* = 6–12 enteroid monolayers/group per experiment. Each symbol indicates an independent monolayer. *p*-values were calculated by one-way ANOVA with Tukey’s post-test for multiple comparisons. *P < 0.05; **P < 0.01; ***P < 0.001; ****P < 0.0001.

The influence of breast milk on Paneth cell protein expression led us to examine its capacity to enhance Paneth cell function, and in particular the production of antimicrobial peptides such as α-defensin 5 (DEFA5), which helps maintain intestinal tolerance and homeostasis (Bevins and Salzman, 2011; Sankaran-Walters et al., 2017). DEFA5 fluorescence intensity was greatly increased in breast milk-treated pediatric monolayers as compared to those treated with infant formula or non-treated controls (Figures 4A,B). Infant formula had no effect on DEFA5 expression. As expected, DEFA5 co-localized with lysozyme-positive Paneth cells (Figure 4B). Surprisingly, increased numbers of a subpopulation of DEFA5-expressing cells that lacked the lysozyme marker was observed in human milk-treated monolayers (Figures 4B,C). Dual DEFA5- and TFF3-positive fluorescent staining revealed co-localization of these two markers, uncovering a breast milk-induced human goblet cell population with capacity to produce DEFA5 (Figure 4D).

We next examined the capacity of breast milk to modulate the production and secretion of cytokines and chemokines typically produced by intestinal epithelial cells. IL-10, IFN-γ, TNF-α, IL-6, IL-8, MCP-1, and GM-CSF were measured in the apical and basolateral milieu of treated and non-treated monolayers. IL-10 and IFN-γ in all conditions were below limit of detection (<0.7 pg). TNF-α and IL-6 were present at very low levels (<1 pg) and below the limit of detection in the non-treated controls, in both apical and basolateral compartments (data not shown). MCP-1, GM-CSF, and IL-8 were detected in apical media and for the most part, levels increased over time (Figures 4E–G). Treatment of pediatric monolayers with infant formula for 72 h resulted in a marked increase of MCP-1 released apically as compared with non-treated monolayers. In contrast, a trend of reduced MCP-1 production was observed upon treatment with human milk (Figure 4E). GM-CSF was produced by untreated monolayers and by those treated with infant formula. In fact, infant formula produced a slight—yet not statistically significant—upregulation of GM-CSF at the 24 h time point (Figure 4F). Conversely, apical GM-CSF secretion was abolished when monolayers were treated with human milk, at both time points tested (Figure 4F). Apical release of IL-8 remained unaffected by treatment (Figure 4G). Basolateral secretion of MCP-1, GM-CSF, and IL-8 was not influenced by treatment either (data not shown). A principal component analysis (PCA) was conducted combing 24 h outcomes described above to visualize, in aggregate, the impact of breast milk and infant formula on epithelial cell physiology (the 24 h time point was selected because it allowed for a complete dataset for all treatments). Monolayers untreated or exposed to infant formula clustered together and were largely distant from those exposed to breast milk by principal component 1 (Figure 4H). Breast milk treatment was associated with biomarkers of enhanced barrier function (DEFA5, occludin, and TER), whereas infant formula was linked to synthesis of pro-inflammatory cytokines (IL-8, MCP-1, and GM-CSF) (Figure 4H).

### Human Milk sIgA Translocates Across Pediatric Enteroid Monolayers

**FIGURE 5:**
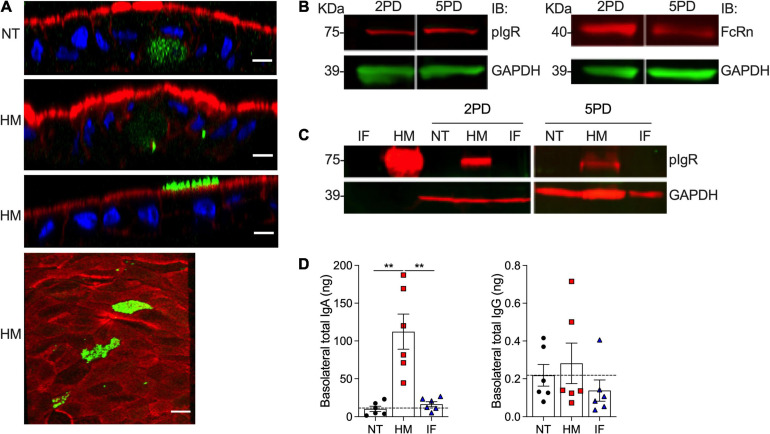
Abundance of pIgR and sIgA translocation in breast milk-treated epithelial monolayers. **(A)** Confocal microscopy images showing SC-pIgR (top, XZ projections, scale bar = 10 μm; bottom, XY projection, scale bar = 5 μm) in 5PD enteroid monolayer NT or treated with 20% (v/v) of HM for 72 h. SC-pIgR, green; actin, red; DNA, blue. **(B)** Composite immunoblotting (IB) showing SC-pIgR and FcRn expression in NT 2PD and 5PD monolayers. **(C)** IB showing the presence of pIgR in HM and its absence in IF, and pIgR staining in 2PD and 5PD monolayers NT or treated for 48 h with 20% HM (v/v) or IF (w/v). **(D)** Total IgA and IgG determined by ELISA in the basolateral media of pediatric monolayers treated with HM and IF as described in panel **(C)**. Data represent mean ± SEM of three experiments, each including two monolayers/group per experiment. Each symbol indicates an independent monolayer. Dashed line indicates limit of detection. *p*-value was calculated by Student’s *t*-test. **P < 0.01.

Breast milk contains a variety of immune mediators, including antibodies that shield immunologically naïve infants from health threats. Maternal immunoglobulins, in particular sIgA, support infant immune development and regulation, enacting long lasting benefits. Early colostrum has high levels of maternal sIgA and IgG, and hence our system enabled us to investigate their interaction with pediatric intestinal epithelial cells. Both 2PD and 5PD monolayers expressed secretory component (SC) of the polymeric immunoglobulin receptor (pIgR) (SC-pIgR), which mediates IgA translocation across the intestinal epithelium as well as the neonatal Fc receptor (FcRn), responsible for transepithelial IgG transport, as shown by immunoblotting (Figures 5A,B). Confocal microscopy images revealed a diffuse cytoplasmic SC-pIgR staining in the non-treated controls, whereas epithelial cells exposed to breast milk exhibited not only intracellular but also a dense apical SC-pIgR staining pattern (Figure 5A). The SC-pIgR increase in both pediatric monolayers treated with breast milk was confirmed by immunoblotting (Figure 5C). Soluble SC-pIgR was detected in breast milk but not in infant formula (Figure 5C). We next examined sIgA and IgG translocation in monolayers treated with breast milk. High levels of sIgA were detected in the basolateral media of monolayers exposed to maternal milk, with mean values substantially higher as compared to media from infant formula and non-treated controls. IgG content was modest and only a trend of higher values was observed in enteroids treated with breast milk as opposed to infant formula or untreated controls (Figure 5D).

### Breast Milk-Induced Protein Upregulation and Basolateral Secretion by Pediatric Epithelial Cells

**FIGURE 6:**
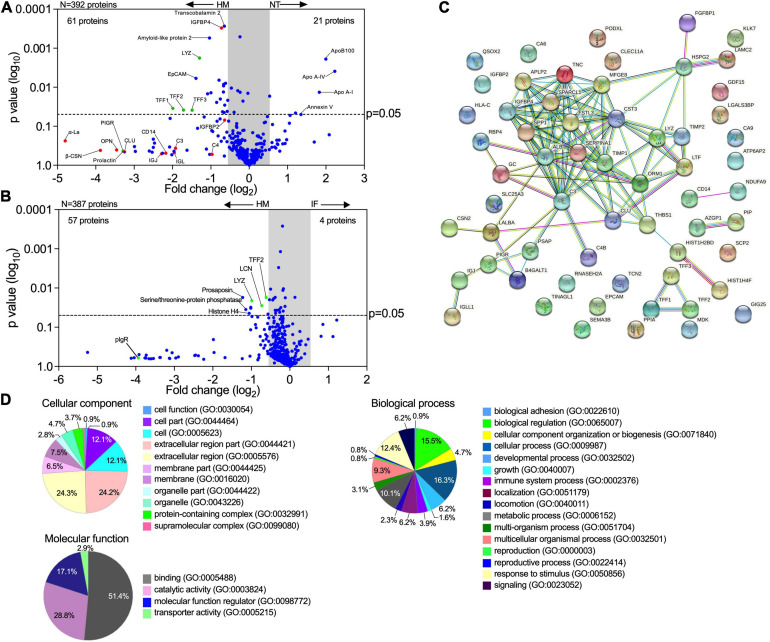
Human milk modifies epithelial cell protein expression and basolateral secretion. **(A,B)** Volcano plots of differential protein abundance (high false discovery rate) in the basolateral culture supernatant of 2PD monolayers NT or treated for 48 h with 20% HM (v/v) or IF (w/v). Data represent protein secreted by up to three monolayers per condition. Red dots indicate HM unique proteins; blue dots indicate epithelial cell-derived proteins; green dots indicate proteins derived from both HM and epithelial cells. **(C)** Protein-protein interaction analysis of 61 upregulated proteins produced by HM-treated monolayers selected based on the cut off shown in panel **(A)**. High confidence interaction score = 0.700. Color line indicates types of protein-protein interactions based on co-expression (black line), co-occurrence (blue line), experimentally determined (magenta line), curated database (light blue line) and textmining search (green line). **(D)** Enrichment analysis of GO terms annotated for cellular component, molecular function, and biological process of the 61 upregulated proteins as described in panel **(A)**.

The intestinal epithelium communicates with underlying tissues *via* secretion of nutrients, growth factors, cytokines, and regulatory peptides. Gut-derived molecules secreted to the basolateral compartment have the potential to disseminate systemically and act on remote tissues, exacting distant modulatory functions. To identify breast milk-induced molecules of intestinal origin that may have a wider (and possibly systemic) impact *in vivo*, we examined proteins secreted into the basolateral compartment of milk-exposed monolayers. Over 15,000 peptide-spectrum matches (PSM) were identified by a proteomic analysis. A total of 392 and 387 secreted proteins with false discovery rate of 1% and PSM ≥ 2 were identified in enteroid monolayers treated with breast milk as compared to monolayers untreated or treated with infant formula, respectively (Figures 6A,B). Applying a *p*-value ≤ 0.05 and log_2_ fold change at ± 0.68 as cutoffs, a total of 61 proteins exhibited increased abundance in the breast milk-treated enteroids as compared to 21 in the non-treated controls (Figure 6A). Similarly, a total of 57 proteins had increased abundance in enteroids treated with breast milk vs. four upon treatment with infant formula (Figure 6B).

Proteins derived from human milk were found in the basolateral compartment of breast milk-treated monolayers, indicating apical to basolateral transepithelial translocation. In addition, we observed increased levels of proteins related to mucosal protection and repair [e.g., TFF1-3, lysozyme C, amyloid-like protein, lipocalin (LCN)], epithelial cell markers (e.g., EpCAM), growth factors [e.g., insulin-like growth factor-binding protein (IGFBP), fibroblast growth factor binding protein (FGFBP)], extracellular matrix remodeling proteins (e.g., metalloproteinase inhibitor proteins, basement membrane-specific heparan sulfate proteoglycan core protein), neurotrophic factor (e.g., prosaposin) and cofactor carrier protein (e.g., transcobalamin 2) in the human breast milk-treated monolayers (Figures 6A,B). In contrast, the non-treated monolayers exhibited increased expression of the apolipoprotein family, and annexin V while infant formula-treated monolayer did not exhibit significant secretion of proteins (Figures 6A,B).

The interactions among proteins with increased abundance in the breast milk-treated vs. non-treated enteroids were examined using the STRING v11.0 database. A theoretical protein-protein network was built based on co-expression and co-occurrence, a curated database, and primary sources for proteins that are commonly associated. A significant protein-protein interaction (*p*-value < 1.0^–16^) was observed among 33 of them (120 edges), whereas 21 proteins showed no interactions within the network (Figure 6C). Additionally, three proteins (TFF family) and two paired proteins [CD14 and ADH dehydrogenase (NDUFA9), and AZGP1 (zinc-α-2-glycoprotein) and PIP (prolactin-inducible protein)] demonstrated specific interaction between each other (Figure 6C). These results indicate that most of the proteins secreted by milk-exposed enteroids do not act as independent entities but can deploy biological activity by either transient or stable association. A functional enrichment analysis was then performed utilizing the PANTHER and AMIGO2 classification database system to highlight the gene ontology (GO) terms annotated for cellular component, molecular function, and biological processes enriched within these protein sets (Figure 6D). The majority of the proteins were associated with the extracellular compartment (24.3%; GO:0044421, GO:0005576) as well as within the cell (12.1%; GO:0044464, GO:0005623) as constitutive protein with cytoplasmic or plasma membrane localization. The main molecular function identified was binding (51.4%; GO:0005488) followed by enzyme activity (28.6%; GO:0003824). In addition, these protein sets participate in multiple biological processes, including cell physiology, response to stimulus, metabolic functions, cell growth and maintenance, and immunity (Figure 6D).

## Discussion

Human breast milk is a rich source of nutrients and bioactive components that promote infant growth and immune development. In this work, using an *ex vivo* pediatric human intestinal stem cell-derived enteroid model, we have identified distinct protein synthesized and cellular functions modulated by human breast milk. HIEs represent a cutting-edge technology that recapitulates the structural and functional features of the human gastrointestinal tissue (Schutgens and Clevers, 2019). They have been used to interrogate gut physiology, host responses to microbes, drug activity, and cell-to-cell communication (King et al., 2018; Co et al., 2019; Chang-Graham et al., 2020; In et al., 2020; Lin et al., 2020; Liu et al., 2020). The health promoting effects of breast milk in the intestinal epithelium have been studied in various suckling animal models. There are, however, fundamental differences in the composition of animal and human milk (Jarvinen et al., 2019), the latter representing a much more complex matrix. Feeding of mice with human breast milk supplemented with specific compounds was employed to identify tolerogenic signals (Ohsaki et al., 2018), and for such specific hypothesis-driven questions, animal studies are valuable. Still, the post-natal maturation timeline of the intestinal epithelium in rodents is substantially different from that of humans (Lim et al., 2020; Stanford et al., 2020). Hence, the data derived from these models has limited relevance for human health (Jarvinen et al., 2019). Others have examined breast milk effects employing human tissue with necrotizing enterocolitis phenotype (Egan et al., 2016; Werts et al., 2020); however, the outcomes reflect disease, not homeostatic intestinal physiology. Our study using pediatric HIE is novel and unique in that interrogates molecular and cellular events influenced by breast milk in a healthy age-relevant human intestinal environment.

A side-by-side comparison of pediatric- vs. adult-derived duodenal HIE monolayers revealed age-associated differences with the former exhibiting shorter columnar epithelial cells and reduced TER, consistent with a less mature epithelial cell phenotype. Reduced enterocyte height has been reported in duodenal biopsies of infants, as compared to adult subjects (Thompson et al., 1998). Together, these results suggest that intestinal epithelial cell development continues through childhood and demonstrate that age-specific cell morphology is preserved in the HIEs.

Several unique molecular events associated with human milk improvement of pediatric intestinal health were observed in our study. The first was the ability of breast milk (colostrum) to enhance epithelial barrier function by reducing ion permeability and upregulating expression of the TJ complex regulator occludin. A recent study reported modulation of TJ gene expression by short-chain fatty acid butyrate, a metabolite that is present in breast milk (Gao et al., 2021). The breast milk-treated monolayers exhibited an unusual pattern of upregulated occludin protein expression. Occludin was detected not only at the (expected) intercellular junctions but also on the apical plasma membranes of absorptive enterocytes as well as Paneth and goblet cells. Condensed occludin-containing vesicles were spread intracellularly. Apical occludin localization has been reported in mouse organoids, primarily in intestinal stem cells and Paneth cells, and less abundantly in enterocytes and goblet cells, and its presence associated with reduced paracellular permeability (Pearce et al., 2018). A regulatory mechanism that involves recruitment of occludin contained in cytoplasmic vesicles or in the apical plasma membrane (via differential phosphorylation) for TJ formation has been proposed (Wong, 1997); under that model, the extra junctional localization may represent protein reservoirs that enable prompt TJ formation required by dynamic metabolic and physiological processes. To the best of our knowledge, ours is the first demonstration of apical and cytoplasmic multi-lamellar occludin expression by human pediatric intestinal cells upregulated in response to breast milk.

A second key observation was the capacity of human milk to substantially increase production of human DEFA5, a peptide that contributes to innate host defense against enteric pathogens and promotes intestinal homeostasis by limiting inflammation and microbial translocation (Salzman et al., 2010; Bevins and Salzman, 2011; Ehmann et al., 2019). DEFA5 was produced not only by Paneth cells (the typical producers of antimicrobial molecules) but also by mucus-producing goblet cells. Production of DEFA5 by intestinal villous TFF3-positive (goblet cells) has been documented in human ileal biopsies (Cunliffe et al., 2001). Our observed expression of DEFA5 in TFF3-positive goblet cells in the pediatric intestine is a new finding and may reflect a differentiating cell lineage stage prompted by breast milk-derived growth factors. Goblet and Paneth cells derive from a common secretory stem cell progenitor under the regulation of ETS transcription factor Spdef (Gregorieff et al., 2009). Lgr5-positive stem cells and Paneth cells are abundant in crypt-like, non-differentiated HIEs. The lifespan of Paneth cells in enteroids is approximately 30 days, regardless of differentiation, as shown in adult differentiated 3D enteroids (Sato et al., 2009). Several bioactive components in human milk including oligosaccharides, lactoferrin, and epidermal growth factor (EGF) have been shown to promote intestinal epithelial proliferation and physiological differentiation in studies using transformed cell lines or animal models (Greene et al., 1986; Buccigrossi et al., 2007; Holscher et al., 2017). Specific elements of breast milk that trigger the cell differentiation events we observed in the pediatric HIE remain to be defined.

The heightening production of TJ proteins and antimicrobial products induced by breast milk (but not infant formula) is consistent with the reported improved epithelial barrier of infants fed with breast milk over those fed by formula as determined by reduced ratio of lactulose-to-mannitol in urine (Catassi et al., 1995).

A third important observation was the immune modulation associated with human milk treatment of pediatric epithelial cells. While infant formula increased the production of pro-inflammatory cytokines MCP-1 and GM-CSF, breast milk reduced MCP-1 levels and totally suppressed apical release of GM-CSF. Gut inflammatory diseases such as inflammatory bowel disease and celiac disease coincide with elevated MCP-1 and GM-CSF in duodenal biopsies (Di Sabatino et al., 2016). Our results illustrate the anti-inflammatory properties of human milk (Hamilton, 2020) in its capacity to reduce or abolish epithelial cell-derived molecules that activate or recruit phagocytic cells and enhance pro-inflammatory cytokines. IL-8, an epithelial cell-derived neutrophil chemoattractant was produced by the pediatric intestinal epithelium. Although not overtly affected by treatment, IL-8 secretion was associated with exposure to infant formula as shown by PCA analysis of early time-point outcomes. This observation underscores the contrasting effects of infant formula vs. breast milk.

IL-8 and MCP-1 are produced copiously by adult enteroids, but not GM-CSF (Noel et al., 2017). Adult HIEs also produce substantial levels of TGF-β1, IFN-γ, and IL-6 (Noel et al., 2017), none of which were detected in the pediatric HIEs described herein. These findings suggest that beyond the immune modulation of maternal milk, the healthy differentiated pediatric intestinal epithelium is intrinsically programmed to silence signals that trigger inflammatory processes.

Human milk’s composition is complex and dynamic, and encompasses a vast diversity of bioactive molecules, reflecting nature’s perfect nutrition, which are absent in infant formula (Ballard and Morrow, 2013). Breast milk soluble components act as prebiotics, antiadhesives and antimicrobials, and include molecules that affect cellular physiology, shield the host from inflammatory and pathogenic insults, and promote healthy gut development (Ballard and Morrow, 2013). Bioactive components with attributed anti-inflammatory and homeostatic function in human milk include IL-10, TGF-β, antioxidants, and enzymes such as lysozyme, glutathione peroxidase, and catalase (Cacho and Lawrence, 2017).

Additionally, human milk contains a variety of growth factor polypeptides (EGF, EGFR ligands, insulin growth factor-I, and transforming growth factor) that participate in tissue maturation and healing (Ballard and Morrow, 2013; Gila-Diaz et al., 2019; Ogra, 2020). Proteomic analyses of human breast milk have been reported elsewhere (Gao et al., 2012; Zhu and Dingess, 2019). We showed herein that many of these milk-derived compounds gain access to the subcellular space.

The exact molecules that trigger the effects described above and operatives, whether they work alone or in a synergistic/complementary manner, still need to be elucidated.

Maternal milk-derived sIgA provides an additional protective immune layer that excludes, neutralizes, and prevents microbial attachment to host cells (Cerutti and Rescigno, 2008). Mucosal dimeric IgA binds to pIgR on the basolateral surface of the epithelial cell membrane and is transported intracellularly and released at the apical surface, carrying a small portion of the pIgR-binding domain (Brandtzaeg, 2013), the SC. Similar mechanism allows for IgM epithelial transport, whereas IgG employs the FcRn to bidirectionally cross epithelial tissues (Pyzik et al., 2015). Maternal antibodies provide antigen-specific defenses, support homeostasis, and promote infant immune development. In animal models, breast milk sIgA conferred long lasting benefits that included maintenance of a healthy microbiota and regulation of epithelial cell gene expression (Rogier et al., 2014).

A fourth relevant finding was the elevation of SC-pIgR in the apical membrane and cytosolic puncta of breast milk-treated enterocytes. Breast milk itself contained an abundance of soluble SC-pIgR, but none was detected in commercial infant formula. The soluble SC-pIgR in maternal milk likely comes from maternal cellular debris. Free SC in human milk can bind enteric pathogens and toxins, and thus boosts non-specific host defenses (Giugliano et al., 1995) in the infant gut. We detected apical-to-basal sIgA transport in the maternal milk-exposed pediatric monolayers. This process supports intracellular pathogen neutralization and delivery of luminal antigens to lamina propria dendritic cells to induce tolerance or subepithelial phagocytic cells to imprint antigen specific immunity (Corthesy, 2013). FcRn detection in the pediatric tissue confirms expression of this receptor beyond infancy. Others have reported FcRn being expressed in human intestinal epithelial cells (Israel et al., 1997; Latvala et al., 2017).

We were unable to detect significant translocation of maternal IgG, despite this process being documented in animal models and cell lines (Dickinson et al., 1999). The variable localization of FcRn and pH requirements may restrict apical-to-basolateral transport while basolateral-to-apical appears to be more prevalent (Aaen et al., 2021). Another explanation could be the lower amount of IgG in human breast milk as compared to sIgA (Czosnykowska-Lukacka et al., 2020). Studies of FcRn distribution, IgG interaction, and IgG immune complex translocation in pediatric HIEs are ongoing.

Beyond promoting a healthy gut, multiple and far-reaching benefits have been attributed to human milk, including prevention of respiratory diseases, immune fitness, cognitive capacity, and overall physiological well-being (Krol and Grossmann, 2018) that endure into adolescence. Breast milk products released to the basal side of the epithelium could, conceivably, distribute systemically and thereby mediate long distant effects. Our proteomic analysis of basolateral media of breast milk-treated monolayers revealed a variety of molecules; some unique to breast milk, such as α-lactalbumin, β-casein, and prolactin, which had evidently translocated across the monolayers, and others that were produced by the milk-exposed pediatric intestinal cells. For the latter, a complex network of interacting biomolecules was revealed, with diverse functions including those affecting growth factors, immune and antimicrobial activity, tissue structure, and homeostasis, which confirms the broad and pleotropic nature of the processes affected by breast milk. The epithelial translocation of milk-derived proteins might have been facilitated by endocytosis of intact (undigested) molecules in our model. These proteins have health benefits by themselves. Milk α-lactalbumin, for example, shields soluble CD14 (sCD14) from proteolytic degradation (Spencer et al., 2010), and sCD14 can bind lipopolysaccharide (LPS) and prevent inflammation and injury caused by soluble LPS or LPS-bearing organisms. β-casein is an immune modulator that regulates cell recruitment, ameliorates inflammation, and stimulates mucus production (Chatterton et al., 2013). Prolactin is a pleiotropic hormone that stimulates production of maternal milk. Expected benefits for the infant, based on animal studies, include reduction of anxiety and stress and neurogenesis (Torner, 2016). In addition, osteopontin prevents inflammation and epithelial damage in mouse DSS-colitis model (Woo et al., 2019).

A variety of breast milk-upregulated tissue-derived proteins were identified, including the TFF family, which maintains and restores gut mucosal homeostasis and regulates complement activation *via* decay-accelerating factor DAF (Andoh et al., 2001); the amyloid-like protein that participates in intestinal metabolic processes and modulates expression of MHC class I molecules (Tuli et al., 2008; Puig et al., 2015); and insulin growth factor binding protein, fibroblast growth factor, basement membrane-specific heparan sulfate protein, and metalloproteinase inhibitor—all of which contribute to epithelial cell growth, tissue development and remodeling, and barrier integrity (Tassi et al., 2011; Austin et al., 2015; Cabral-Pacheco et al., 2020). Other secreted proteins included transcobalamin 2, which facilitates the transport of vitamin B12 within the organs (Quadros et al., 1999) and epithelial cell adhesion molecule (EpCAM), which localizes in the basal cell membrane and facilitates cell-to-cell interaction and proliferation (Das et al., 2019). Complement proteins (C3 and C4) were also present in the basal media from breast milk-treated enteroids; C4 participates in complement activation *via* the classical and lectin pathway, whereas C3 is a converging substrate for all activating pathways; C3 cleavage into C3a and C3b, along with C5 cleavage, triggers the rest of the complement cascade. C3, C4, and other complement components are present in human breast milk (Gao et al., 2012; Zhu and Dingess, 2019). Likewise, human intestinal epithelial cells produce complement proteins (Moon et al., 1997; Kopp et al., 2015). The origin of the complement proteins we identified is unclear. We surmise they derive from breast milk because synthesis of complement proteins by the intestinal epithelial cells reportedly requires pro-inflammatory signals (downregulated by breast milk in our system) (Andoh et al., 1993). Nonetheless, the fact that maternal complement molecules would trespass the pediatric epithelium is intriguing. Regardless of their source, complement can boost infant mucosal protective mechanisms (Ogundele, 2001). Bovine colostrum has been shown to influence the proteome of HT-29 cells as well as epithelial cell glycosylation (Morrin et al., 2019). We show, for the first time, that human milk influences the synthesis of multiple mediators of metabolic and physiologic functions that act locally or systemically.

The proteomic data was consistent with our other observations. For instance, TFF and Lyz, which were abundant in the secretome of breast milk treated enteroids, were also upregulated in Paneth and goblet cells, respectively, as revealed by confocal microscopy. The functional validation of other relevant proteins warrant future studies; our findings are hypothesis-generating and stimulate further investigation.

In contrast to the abundant protein content of enteroid monolayers treated with human breast milk, treatment with infant formula did not exhibit an increased protein profile compared to untreated controls. These results emphasize the unique capacity of breast milk in contributing molecules that support host organogenesis and tissue homeostasis, immunity and many other biological processes, all of which were absent in tissue exposed to infant formula.

In summary, using a novel *ex vivo* pediatric HIE, several molecular and cellular events associated with breast milk that improve intestinal health were identified: (1) cell differentiation and strengthening of the pediatric intestinal epithelial barrier by reduction of ion permeability, increase of epithelial cell height, and upregulation of TJ occludin with a unique expression pattern; (2) boosting of innate immunity by enhanced production of antimicrobial DEFA5 by Paneth and goblet cells; (3) immune modulation and passive immunization by increase of pIgR and translocation of luminal sIgA; (4) reduction of pro-inflammatory cytokines; (5) translocation of breast milk proteins with anti-inflammatory and anti-microbial properties; and (6) expression of proteins responsible for tissue remodeling, healing, and mucosal homeostasis by epithelial cells.

The 2D pediatric HIE model we described is a practical and powerful tool to investigate nutrients, bioactive molecules, and therapeutic and disease preventing agents that can improve childhood health.

## Data Availability Statement

The datasets presented in this study can be found in online repositories. The names of the repository/repositories and accession number(s) can be found below: http://www.proteomexchange.org/, PXD025966.

## Ethics Statement

The studies involving human participants were reviewed and approved by Johns Hopkins University School of Medicine (IRB) NA 00038329 and University of Maryland School of Medicine (IRB) HP-00065842. The patients/participants provided their written informed consent to participate in this study.

## Author Contributions

GN, JGI, and JML-D conducted the experiments and analyzed the data. JML-D compiled the final figures. LRD and RNC conducted the proteomics analysis. ALG obtained the pediatric biopsies. JDC obtained the human milk samples. OK and MFP conceptualized the study, secured the funding, and designed the experiments and data analysis. All authors contributed to the writing and editing of the manuscript.

## Conflict of Interest

The authors declare that the research was conducted in the absence of any commercial or financial relationships that could be construed as a potential conflict of interest.

## Footnotes

1 www.matrixscience.com
